# Aberrant regulation of the LIN28A/LIN28B and let-7 loop in human malignant tumors and its effects on the hallmarks of cancer

**DOI:** 10.1186/s12943-015-0402-5

**Published:** 2015-06-30

**Authors:** Tianzhen Wang, Guangyu Wang, Dapeng Hao, Xi Liu, Dong Wang, Ning Ning, Xiaobo Li

**Affiliations:** Department of Pathology, Harbin Medical University, Harbin, China; Department of Gastrointestinal Medical Oncology, The Affiliated Tumor Hospital of Harbin Medical University, Harbin, China; Faculty of Health Sciences, University of Macau, Taipa, Macau, China; Center of Cardiovascular, Inner Mongolia People’s Hospital, Hohhot, Inner Mongolia China; College of Bioinformatics Science and Technology, Harbin Medical University, Harbin, China; Department of Gastrointestinal Surgery, International Hospital of Pecking University, Beijing, China

**Keywords:** RNA binding proteins, MicroRNAs, LIN28A/LIN28B and let-7 loop, Hallmarks of cancer

## Abstract

RNA binding proteins (RBPs) and microRNAs (miRNAs) are two of the most important post-transcriptional regulators of gene expression, and their aberrant expression contributes to the development of human malignancies. Let-7, one of the most well-known tumor suppressors, is frequently down-regulated in a variety of human cancers. The RBP LIN28A/LIN28B, a direct target of the let-7 family of miRNAs, is an inhibitor of let-7 biogenesis and is frequently up-regulated in cancers. Aberrant regulation of the LIN28A/LIN28B and let-7 loop in human malignant tumors is reportedly involved in cancer development, contributing to cellular proliferation, cell death resistance, angiogenesis, metastasis, metabolism reprogramming, tumor-associated inflammation, genome instability, acquiring immortality and evading immune destruction. In this review, we summarized the mechanisms of LIN28A/LIN28B and let-7 loop aberrant regulation in human cancer and discussed the roles and potential mechanisms of the LIN28A/LIN28B and let-7 loop in regulating the hallmarks of cancer. The crosstalk between LIN28A/LIN28B and let-7 loop and certain oncogenes (such as MYC, RAS, PI3K/AKT, NF-κB and β-catenin) in regulating hallmarks of cancer has also been discussed.

## A double-negative feedback loop between LIN28A/LIN28B and let-7

MicroRNAs (miRNAs) are small non-coding RNAs that bind the mRNA of target genes to inhibit their translation and/or induce their decay. MicroRNAs thus play a crucial role in many biological events, including tumorigenesis. Briefly, most miRNAs are transcribed by RNA polymerase II [1]. Primary miRNA transcripts (pri-miRNAs) are then processed in the nucleus by the RNAseIII Drosha into 70-100-nt-long pre-miRNAs, which are then exported to the cytoplasm and cleaved by the RNAse III Dicer to form ~22-nt-long dsRNAs (miRNA). Finally, the RNA-induced silencing complex (RISC) binds to one strand of the dsRNA and guides it to target mRNA for subsequent silencing [2].

The miRNA let-7 was identified in the nematode Caenorhabditis elegans in 2001, seven years after let-4, the first known miRNA, was identified in the same species [3]. The let-7 family of miRNAs is the largest of all miRNA families, and members of this family are highly conserved in sequence and function from C. elegans to humans [4, 5]. It’s now known that members of let-7 family play important roles in regulating cellular differentiation, metabolism and the development of certain diseases, including tumorigenesis [6].

The highly conserved RNA binding proteinLIN28 family includes two homologous members, LIN28A and LIN28B, each having similar domain structure and function. Like let-4 and let-7, LIN28A was also first identified in C.elegans [7], though it is also present in a wide variety of mammals. Notably, LIN28A gene mutation in C. elegans results in disturbance of its developmental timing [8]. LIN28B was first identified in hepatocellular carcinoma, where levels of the protein were high [9]. Recent studies found that LIN28A/LIN28Band let-7 family miRNAs tend to have opposing roles in many cellular processes, in particular those involved in cancer development and progression [10]. Indeed, LIN28A/LIN28B and let-7 are inversely expressed in normal and malignant tissues [11, 12]. The presence of a double-negative feedback loop between LIN28A/LIN28B and let-7 was also reported [10].

**Fig. 1:**
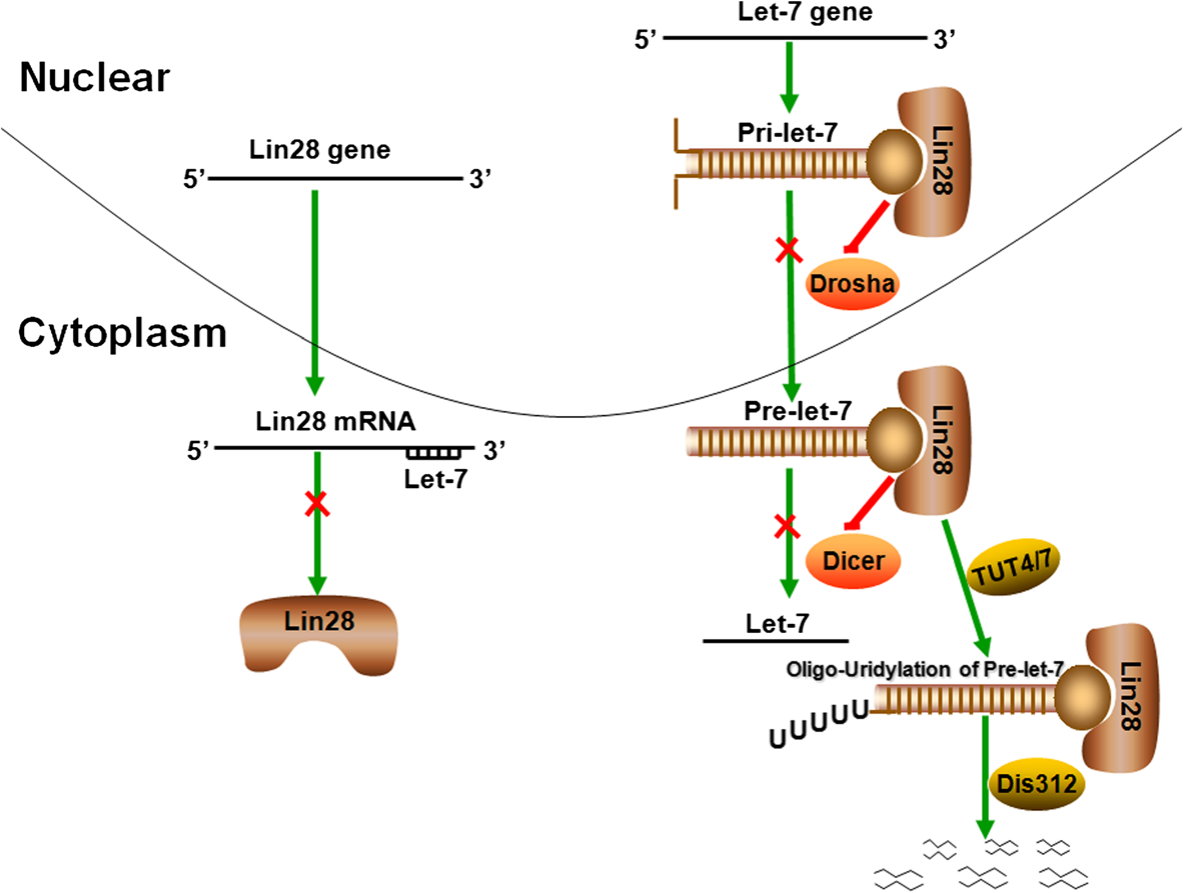
A double-negative feedback loop between LIN28A/LIN28B and let-7

LIN28A/LIN28B negatively regulates let-7family miRNAs via its RNA-binding domains (RBDs), which include a cold-shock domain (CSD) at the N-terminus and two Cys-Cys-His-Cys (CCHC)-type zinc finger domains at the C-terminus [13–16]. Both the CSD and CCHC zinc fingers of LIN28A/LIN28B can interact with the conserved residues ofpri-let-7 and pre-let-7. Briefly, the CSD inserts into the apical point of the precursor loop, while the CCHC zinc fingers dimerize on a GGAG motif adjacent to the Dicer cleavage site [17, 18]. The binding of LIN28A/LIN28B to either pri-let-7 or pre-let-7 inhibits let-7 precursor processing by Drosha and Dicer [19]. Upon binding to pre-let-7, LIN28A/LIN28B recruits TUT4/TUT7, which causes oligo-uridylation at the 3′terminal of pre-let-7 [20–22]. Under normal conditions, Dicer recognizes the two-nucleotides at the 3′ terminal via its PAZ domain; however, oligo-uridylation elongates the 3′ terminal resulting in resistance to Dicer cleavage. Oligo-uridylated pre-let-7 can also be degenerated by the 3′-5′ exonuclease Dis312 [23, 24]. Thus, LIN28A/LIN28B not only inhibits the biogenesis of let-7 family miRNAs, but also induces their degradation. Conversely, let-7 miRNA may bind complementary sites on the 3′ UTR of both LIN28A and LIN28B mRNAs, thus inhibiting the expression and function of LIN28A/LIN28B protein [9, 25]. This double-negative feedback loop between LIN28A/LIN28B and let-7 is shown in Fig. 1.

## The mechanisms of aberrant expression of LIN28A/LIN28B and let-7 in cancer

LIN28A/LIN28B proteins are frequently up-regulated in various malignancies originating from three germ layers (Table 1). High levels of LIN28A/LIN28B proteins are associated with many cancer biological behaviors and poor prognosis.

**Table 1:** Pathological associations of increased LIN28A/LIN28B and or of decreased let-7 expression in various cancer tissues

Origin	Cancer type	Ref.	Pathological association	
Endoderm	Colon	LIN28↑	[12, 74]	Increased tumor progression and metastasis
		Let-7↓	[113, 114]	Poor prognosis
	Lung	LIN28↑	[12, 115]	Increased proliferation
		Let-7↓	[116]	Poor prognosis; increased cellular proliferation
	Hepatocellular carcinoma	LIN28↑	[12, 117]	Advanced-stage cancer
		Let-7↓	[118]	Metastatic cancer; increased proliferation and migration
	Gastric adenocarcinoma	LIN28↑	[119]	Poor prognosis
		Let-7↓	[76]	Increased invasion and metastasis
	Esophageal	LIN28↑	[75]	Increased proliferation and metastasis
	Pancreatic	Let-7↓	[120]	Increased proliferation
Mesoderm	Cervical	LIN28↑	[12]	
	Ovarian	LIN28↑	[12, 121]	High-grade cancer
		Let-7↓	[121, 122]	High-grade cancer
	Germ cell tumor	LIN28↑	[123]	Poor prognosis
	Prostate	LIN28↑	[124]	Increased proliferation
		Let-7↓	[125]	Increased proliferation
	Chronic myeloid leukemia	LIN28↑	[12]	Increased tumor progression
	Burkitt lymphoma	Let-7↓	[126, 127]	Increased proliferation
	Renal cell carcinoma	Let-7↓	[128]	Metastatic and high-grade cancer
Ectoderm	Breast	LIN28↑	[12, 55, 129]	Increased tumor aggressiveness and proliferation
		Let-7↓	[81]	Lymph node metastasis
	Oral squamous cell carcinoma	LIN28↑	[90]	Poor prognosis
	Glioma	LIN28↑	[130]	Increased proliferation
	Melanoma	Let-7↓	[131]	Increased invasion

LIN28A/LIN28B transactivation by various transcription factors in malignancies has been extensively studied. LIN28B may be up-regulated via direct promoter binding by the transcription factor c-myc upon activation of the MAPK signaling pathway [26] or by NF-κB during inflammation [27]. LIN28B may also be up-regulated by STAT3 during inflammation-mediated epithelial-to-mesenchymal transition (EMT) [28] or by β-catenin upon activation of the Wnt signaling pathway [29]. The transcriptional factor SOX2 reportedly up-regulates LIN28A by binding to a proximal site on the promoter and facilitating promoter acetylation via interaction with the histone acetyltransferase complex [30].

LIN28A/LIN28B is also regulated post-transcriptionally. In addition to let-7, the miRNAs miR-26a, miR-181, miR-9, miR-30, miR-125, miR-212 and miR-27 have also been shown to directly bind the 3′UTR of LIN28A/LIN28B and repress translation of the protein, and as these miRNAs are frequently under-expressed in malignant tumors, higher levels of LIN28 expression are seen [31–34]. Notably, a potential regulatory loop reportedly exists between LIN28B and miR-212 in androgen-independent prostate cancer [35]. A recent study revealed that LIN28A/LIN28B mRNA contains an AU-rich element (ARE) within its 3′UTR, and the tumor suppressor tristetraprolin (TTP), an ARE binding protein, enhances the degradation of LIN28A/LIN28B mRNA; however, TTP is often repressed in human cancers, which may also contribute to the elevation of LIN28A/LIN28B in certain cancer types [36]. The ribonuclease DIS3, one of the most frequently mutated genes in multiple myeloma, is an inhibitor of LIN28B through binding and degrading LIN28B mRNA [37]. More recent research revealed that the expression of insulin-like growth factor 2 (IGF2) mRNA-binding protein 3 (IMP3), a protein which regulates RNA localization, translation and stability, correlates with that of LIN28B and cytoplasmic IMP3 granules contain LIN28B mRNA. Further studies showed that IMP3 recruits LIN28B mRNA and prevents the binding of argonaute 2 (Ago2) and let-7 to LIN28B, thus allowing the increased expression of it and other let-7 target genes, like HMGA2 [38]. Additionally, the protein level of LIN28B has been revealed to be regulated via ubiquitin-mediated proteasomal degradation. The human TRIM-NHL domain-containing protein TRIM71, an ubiquitin ligase, was reported to negatively regulate the stability of LIN28B protein by catalyzing its polyubiquitination [39]. However, weather the regulation of LIN28B protein is altered in malignancies is still waiting for further experiments to reveal.

In contrast to the expression of LIN28A/LIN28B proteins, the expression of let-7 family miRNAs is typical decreased in cancers (Table 1). While let-7 miRNAs may be regulated at multiple levels, most studies support the significance of their post-transcriptional regulation. For instance, during tumorigenesis, mature let-7 was found to be absent, whereas pri-let-7 was present at high levels, which suggests post-transcriptional regulation of mature let-7 [40]. As previously mentioned, LIN28A/LIN28B is a common post-transcriptional repressor of let-7 miRNAs. In addition to LIN28A/LIN28B proteins, the complex of NF90 and NF45 proteins can inhibit pri-let-7a processing into pre-let-7a by binding to pri-let-7a [41], while Ago proteins can bind and stabilize mature miRNAs and thereby increase let-7 levels [42]. Importantly, as the endonucleases Drosha and Dicer are essential for the processing of miRNA to maturation, factors influencing the activity or expression of these endonucleases impact the processing of miRNAs. For instance, Fas and TRAIL-R2 were reported to reduce the levels of mature let-7 miRNA by inhibiting the activities of Dicer [43] and Drosha [44], respectively.

Regulation of let-7 expression also occurs at the transcriptional level. Notably, the presence of a CpG island in the promoter region of let-7a-3, located on chromosome 22q13.31, allows for epigenetic regulation via DNA methyltransferases DNMT1 and DNMT3B [45, 46]. The nuclear hormone receptor DAF-12, a transcriptional activator or repressor depending on the presence or absence of a DA (dafachronic acid) ligand, can directly modulate the transcription of certain let-7 miRNAs [47]. Conversely, let-7 miRNAs can repress DAF-12 expression by binding its 3′UTR, which suggests a complex feedback loop between DAF-12 and let-7 miRNAs [48]. In addition to being repressed for their expression, the antitumor functions of let-7 have also been attenuated in malignant tumor cells. Competing endogenous RNA (ceRNA) is a hypothesis driven by the reasoning that mRNA, transcribed pseudogenes and long non-coding RNA (lncRNA) compete for a limited pool of miRNAs [49]. Even though the hypothesis of ceRNA is challenged by some researchers recently [50], ceRNAs attenuating let-7-mediatedantitumor activity has been extensively reported. For example, the lncRNAH19 reportedly inhibits the bioavailability of let-7 family miRNAs through a molecular sponge mechanism [51]. A recent study also found that high-mobility group A (HMGA2), a non-canonical transcriptional factor, promoted lung cancer progression independent of its protein-coding function. Indeed, HMGA2 functions as a ceRNA, competing with the transforming growth factor beta receptor 3(TGFBR3) for let-7, thus allowing for the heightened expression of TGFBR3 and subsequent lung cancer progression [52].

As discussed, the expression patterns and functions of LIN28A/LIN28B and let-7 in malignancies are largely opposing and appear to compose a double-negative feedback loop regulating cancer progression.

## LIN28A/LIN28B and let-7 loop regulates the hallmarks of cancer

Hanahan and Weinberg famously described ten biological capabilities acquired by cancer during development [53]. These include: sustaining proliferative signaling, resisting cell death, evading growth suppressors, inducing angiogenesis, enabling replicative immortality, activating invasion and metastasis, accumulating genome instability, inducing inflammation, reprogramming of energy metabolism and evading immune destruction [53]. To date, the LIN28A/LIN28B and let-7 loop has been demonstrated to regulate almost all of these hallmarks.

### LIN28A/LIN28B and let-7 loop regulates cancer cell proliferation

One of the most fundamental characteristics of cancer cells is their capacity for uncontrolled proliferation. Unlike normal cells, whose proliferation is strictly controlled to maintain homeostasis, cancer cells have developed the ability to sustain proliferative signaling, therein becoming masters of their own destinies [53]. Many studies of multiple cancer types have shown that LIN28A/LIN28B promotes the proliferation of cancer cells through five different mechanisms: up-regulation of cell cycle regulators, elevation of cellular proliferative signaling, activation of proliferation-associated transcription factors, facilitation of ribosomal protein synthesis and activation of cellular metabolism.

LIN28A/LIN28B has been demonstrated to up-regulate cell-cycle regulators in two ways. First, LIN28A/LIN28B directly binds and promotes the translation of numerous mRNAs encoding cyclins (cyclinA, cyclinB and cyclinD), cyclin-dependent kinases (CDK1, CDK2 and CDK4) and cell division cycle proteins (CDC2 and CDC20) [54]. Secondly, through repressing let-7, LIN28A/LIN28B indirectly up-regulates some cell-cycle regulators targeted by let-7, such as cyclinD1/2, CDK6, CDC34, CDC25a and Trim71 (a repressor of CDK inhibitor 1A).

LIN28A/LIN28B can elevate cellular proliferation signals in both let-7-dependent and -independent manners. Through inhibiting let-7, LIN28A/LIN28B can activate a variety of cellular proliferation signaling pathways. For instance, let-7 targets the IGF1 receptor and AKT2 to inhibit PI3K/AKT pathway activity and RAS to inhibit MAPK pathway activity. Thus, inhibition of let-7 by LIN28A/LIN28B would increase the activities of both pathways and, subsequently, increase proliferation. As a RNA binding protein, LIN28A/LIN28B also directly binds to and promotes the translation of IGF2. Recently, it was also found that LIN28A/LIN28B promotes the expression of human epidermal growth factor receptor 2 (HER2) at the post-transcriptional level in breast cancer cells [55].

Activation of transcriptional factors necessary for cellular proliferation in a let-7-dependent manner is another method by which LIN28A/LIN28B can increase proliferation. For instance, hepatitis B virus x protein (HBx) promotes cellular proliferation through down-regulating let-7 expression, thus elevating levels of the transcription factor signal transducer and activator of transcription 3 (STAT3), another let-7 target, in HBV infected cells [56]. Additionally, let-7 represses the proliferation of cancer cells by directly targeting HMGA2, a protein which is frequently over-expressed in and promotes proliferation of many cancer types [52, 57, 58].

LIN28A can also increase cellular proliferation through directly binding to and promoting the translation of numerous mRNAs encoding ribosomal proteins, such as RPS13, EEF1G and EIF4A [59]. Additionally, through the LIN28A/LIN28B-mediated inhibition of let-7, PI3K/AKT-mTOR signaling may promote ribosomal biogenesis and translation in mammary cells via activating S6, eIF4E and eIF4B, as let-7 is known to target key components of this pathway, such as AKT2 and Raptor [60, 61]. A detailed discussion of the LIN28A/LIN28B-mediated activation of cellular metabolism and subsequent promotion of cellular proliferation is presented in the next section.

Of note, under certain conditions, LIN28A/LIN28B may also inhibit cancer cell proliferation. Indeed, Song et al. reported that over-expression of LIN28A/LIN28B in gastric cancer cell line BGC-823 inhibited proliferation through some unknown mechanism [62]. However, as the authors used only one cell line, whether LIN28A/LIN28Btruly inhibits proliferation of gastric cancer cells is still not clear. Moreover, since extensive elevation of oncoproteins, such as RAS, MYC and RAF, can induce cell senescence and/or apoptosis [53], the reported inhibition may have been the result of cell senescence triggered by extensive proliferative signals.

### LIN28A/LIN28B and let-7 loop regulates cancer cell metabolism

Metabolic shift is a basic property of cancer cells. In the 1920s, Otto Warburg discovered that glycolysis was maintained in cancer cells in conditions of high oxygen tension, otherwise known as “aerobic glycolysis”. During enhanced glucose uptake and elevated glycolysis, intermediates of the glycolytic pathway become a major resource for anabolic reactions in cancer cells. For example, dihydroxyacetone phosphate is important for synthesis of triacylglycerides and phospholipids, glucose 6-phosphate is necessary for the synthesis of glycogen and ribose 5-phosphate, and pyruvate is an important progenitor of amino acids and may enter a truncated tricarboxylic acid (TCA) cycle and generate acetyl-CoA, which is necessary for the synthesis of fatty acids, cholesterol and isoprenoids. Thus, through augmenting anabolic reactions, glycolysis is a promoter of cancer cell growth and proliferation [63]. Both LIN28A and LIN28B reportedly enhance aerobic glycolysis, while let-7 suppresses this process at least in part through targeting pyruvate dehydrogenase kinase 1(PDK1), which negatively regulates pyruvate dehydrogenase (PDH), thus preventing pyruvate entry into TCA under normoxic conditions [64]. LIN28A/LIN28B also directly potentiates cellular metabolism through binding and regulating translation of glycolysis enzymes such as hexokinase 1 (HK1), pyruvate dehydrogenase alpha 1 (PDHA1) and PDHB [59].

**Fig. 2:**
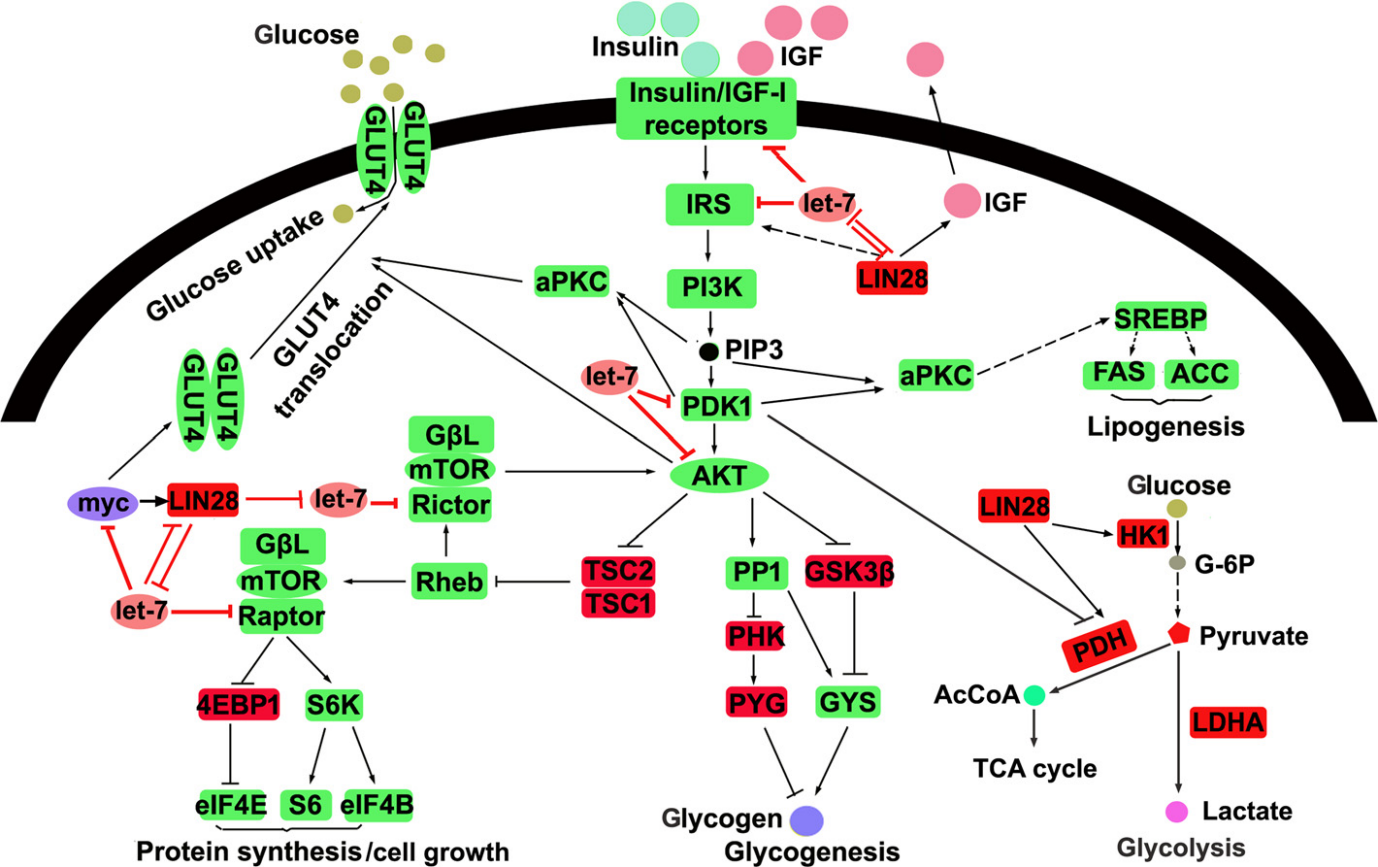
Let-7 targets insulin signaling pathway and thus inhibits cancer cell metabolism

Insulin signaling is a master regulator of cellular anabolic metabolism [65]. By activing the PI3K/AKT pathway through binding insulin receptors, insulin not only facilitates glucose uptake and promotes glycogenesis, but also promotes protein synthesis and lipogenesis. Thus, insulin-PI3K/AKT signaling is a key regulator coupling cellular anabolic metabolism with cellular growth and proliferation. Through let-7, LIN28A/LIN28B activates insulin signaling by elevating components involved in insulin signaling pathways, such as IGF1R, insulin receptor (InsR), IRS2, AKT2 and Rictor (Fig. 2) [60]. LIN28A/LIN28Balso directly activates insulin signaling through binding and activating translation of components and regulators of insulin signaling pathways, such asIGF2 and HMGA1. IGF2 is a ligand of InsR, and the interaction between IGF2 and InsR triggers the activation of insulin signaling. HMGA1 is a key regulator of the transcription of InsR [66].

### LIN28A/LIN28B and let-7 loop mediates cancer cell evasion of immune destruction

The immune system is responsible for recognizing and eliminating cancer cells; however, tumors typically evade immune destruction through either avoiding detection by the immune system or limiting the extent of immunological eradication [53]. Recent studies suggest that the LIN28A/LIN28B and let-7 loop may also regulate cancer cell immune evasion.

The transmembrane protein Fas (CD95) is a member of the tumor necrosis factor (TNF) receptor superfamily. Binding of either Fas ligand (Fas-L), a type II transmembrane protein expressed on cytotoxic T lymphocytes, or TNFα, a cytokine secreted by activated macrophages and other immune cells, induces trimerization of Fas in the membrane of the target cell (including cancer cells) and results in the activation of Fas. Fas activation then leads to the activation of caspase 8, triggering extrinsic apoptosis [67]. Let-7 expression has been shown to decrease during Fas-mediated apoptosis because Fas activation suppresses Dicer; however, exogenous expression of let-7 inhibits cell sensitivity to Fas-mediated apoptosis via directly targeting Fas [43, 68], which suggests that let-7 family miRNAs may suppress tumor innate immune reactions.

Toll-like receptors (TLRs) are a class of transmembrane proteins expressed in macrophages, neutrophils, dendritic cells and other immune cells, and play a key role in innate immune response via recognizing inflammatory mediators, costimulatory molecules and even conserved structures of microbes. Peptides, lipopolysaccharides and nucleic acids may each act as TLR ligands [69]. TLRs are universally expressed in many cancer types and promote the development of inflammation-associated malignances through activating the inflammatory response. However, TLRs have also been shown to be sensors of cell death, and stimulation of TLRs to activate the innate immune system is a strategy currently under development for cancer therapy [69]. A recent study uncovered that extracellular let-7 interacts with and then activates TLR7, an RNA-sensing neuronal TLR, and induces neurodegeneration [70]. Interestingly, in a metastatic gastric cancer cell line, let-7 family miRNAs could be selectively secreted into the extracellular environment via exosomes [71]. These results suggest that the activation of TLR7 induced by extracellular let-7 may also be involved in the regulation of immune response or inflammation in cancer; however, this hypothesis has yet to be validated experimentally.

### LIN28A/LIN28B and let-7 loop mediates tumor-associated inflammation

**Fig. 3:**
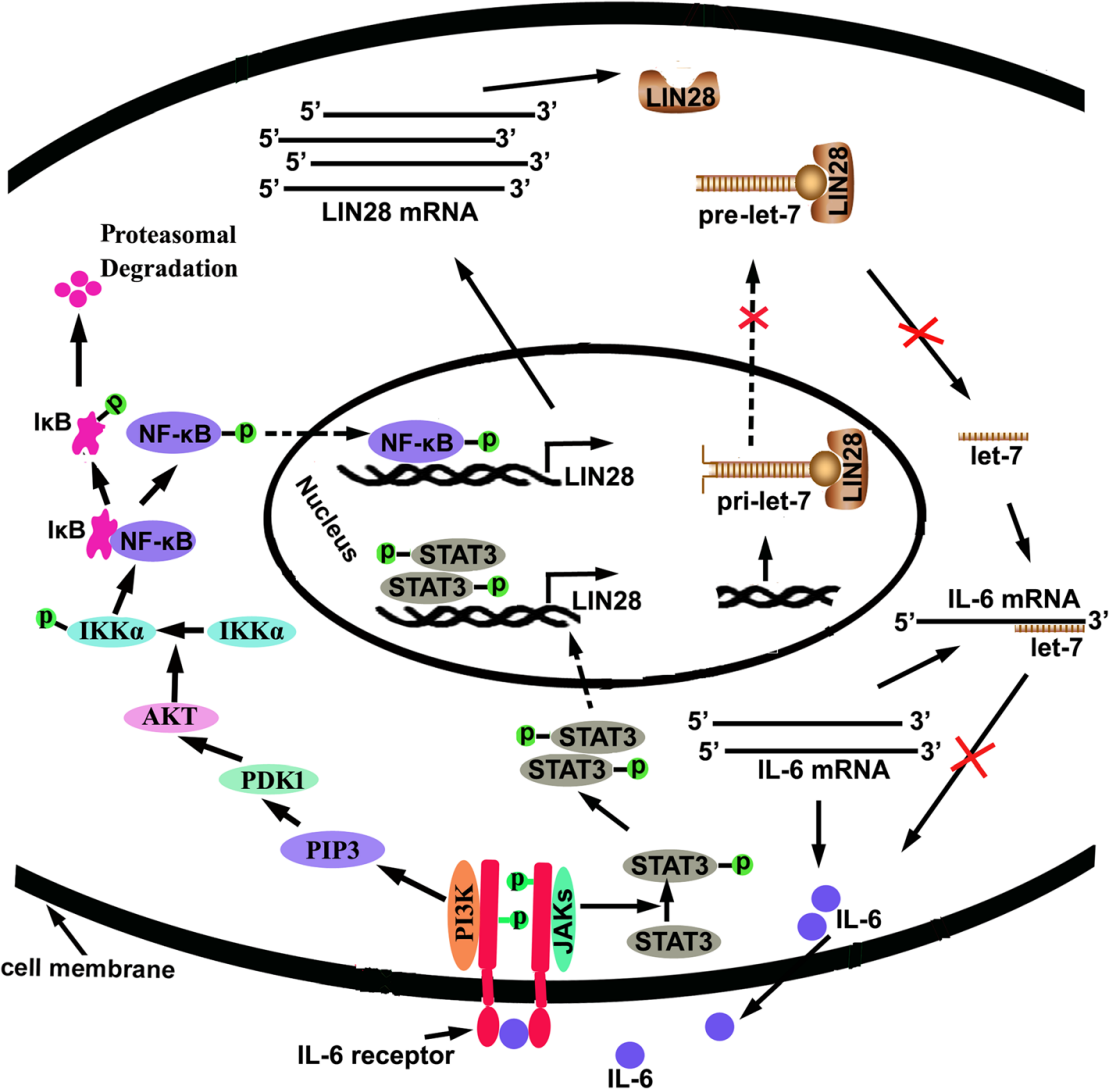
A positive feedback between LIN28A/LIN28B and transcriptional factor NF-κB and STAT3 in the process of inflammation mediated cancer progression

Inflammation is linked clinically and epidemiologically to cancer. However, the molecular intersections between inflammation and cancer progression have been unclear for a long time. Recently, it was demonstrated that the LIN28A/LIN28B and let-7 loop is a key switch linking inflammation to cell transformation. Viswanathan et al. were the first to evaluate the role of LIN28A in cell transformation, over-expressing LIN28A in NIH/3 T3 cells [12]. They observed that LIN28A over-expression promoted 3 T3 cells to form clones in vitro and form solid tumors in nude mice with a concomitant down-regulation of multiple mature let-7 family member miRNAs. Importantly, this effect could be attenuated by re-introducing let-7. Recently, a consistent result was observed by Madison et al. in intestinal epithelial cells. They showed that targeted expression of LIN28B promoted crypt transformation and fostered intestinal polyp and adenocarcinoma formation in vivo in a let-7-dependent manner [72]. In revisiting the molecular intersection between inflammation and cancer progression, Dimitrios and colleagues revealed such an intersection between inflammation and cell transformation [27]. They showed that over-expression of LIN28B upon the activation of NF-κB inhibited the generation of let-7 family member miRNAs and elevated the production of IL-6, a target of let-7. In turn, IL-6 activated NF-κB and STAT3 transcription factors through the RTK signaling pathway. The activation of NF-κB and subsequent production of IL-6 thus formed a positive feedback loop (Fig. 3), while STAT3 activation is necessary for the transformation of normal cells. Furthermore, they showed that STAT3 directly activated miR-181b and miR-21 at the transcriptional level. MiR-181b and miR-21 target cylindromatosis (CYLD) and phosphatase and tensin homolog (PTEN), respectively, and down-regulation of CYLD and PTEN leads to NF-κB activation, therefore also acting as a part of the epigenetic switch linking inflammation to cancer [73]. As previously mentioned, STAT3 also suppresses the expression of let-7 through directly activating LIN28A/LIN28B expression during inflammation-stimulated EMT [28].

### LIN28A/LIN28B and let-7 loop regulates metastasis

Local invasion and distant metastasis are marks of higher pathological stages of malignant cancers. While the invasion-metastasis cascade is known to consist of a succession of processes, beginning with local invasion and followed by intravasation (cancer cell invasion into blood and lymphatic vessels) and extravasation (cancer cell escape from vessels) to form micrometastases and finally grow into macroscopic tumors, the mechanisms involved in this multistep process are still being defined. Of all the steps, however, local invasion is the most extensively studied.

EMT is broadly believed to regulate invasion [53]. EMT is characterized as epithelial cells losing their cell polarity and cell-cell adhesion with the loss of E-cadherin expression usually driven by the elevated expression of a set of transcriptional factors, such as Snail, Slug, Twist and Zeb1/2 [53]. Involvement of the LIN28A/LIN28B and let-7 loop in the regulation of cancer cell invasion and metastasis is, naturally, intimately associated with EMT. Many studies have shown that LIN28A/LIN28B promotes and let-7 inhibits invasion and metastasis in various cancer types, including colon cancer, breast cancer, hepatocellular carcinoma, pancreatic cancer, gastric cancer, lung cancer and esophageal cancer [57, 74–79]. In fact, the mechanism by which let-7 inhibits invasion and metastasis is, actually, well-studied. HMGA2 is the most frequently reported target of let-7 in the process of inhibiting invasion and metastasis [57, 77]. HMGA2 has been demonstrated to promote EMT by inducing the expression of Slug and Snail and then inhibiting the expression of E-cadherin in many cancer types [57, 80]. In addition to regulating invasion and metastasis via a coding gene, HMGA2 also functions as a ceRNA to facilitate cancer metastasis in certain cancer types [52]. As previously mentioned, by competing with TGFBR3 to bind let-7, HMGA2 represses the inhibitory effect of let-7 on TGFBR3, thus elevating TGFBR3protein and facilitating cancer invasion and metastasis [52]. BesidesHMGA2, let-7 also was reported to inhibit invasion, migration and metastasis via targetingITGB3, MAP4K3 and MYH9 [76, 79]. A recent study showed that let-7 inhibited the cancer cell migration via direct targeting of four genes in the actin cytoskeletal pathway, including RDX, DIAPH2, ITGB8 and PAK1 [81]. IL-6 was also a direct target of let-7 to inhibit cancer cell invasion and migration. It was reported that down-regulation oflet-7 in cancer-associated mesenchymal stem cells (MSCs) results in the enhanced secretion of IL-6, and IL-6 then promotes prostate cancer cell metastasis [82]. LIN28A/LIN28B promotes invasion and metastasis through the let-7/HMGA2/Slug or Snail/E-cadherin axis [57, 77], but also in a let-7-independent manner. LIN28A directly binds and promotes the translation of HMGA1, and like HMGA2, HMGA1 promotes EMT by inducing the expression of Slug and Snail. Additionally, LIN28A has been found to directly bind E-cadherin mRNA and repress the translation of E-cadherin mRNA in embryo stem cells [83]; however, the role of LIN28Ain directly regulating E-cadherin expression in cancer cells is still unclear.

### LIN28A/LIN28B and let-7 loop regulates cancer cell death

The LIN28A/LIN28B and let-7 axis is known to regulate cellular apoptosis and is involved in resistance/sensitivity to therapy. Many studies have shown that the over-expression of let-7 or knockdown of LIN28A/LIN28B increases the radiosensitivity or chemosensitivity of cancer cells [84–87]. Let-7 reportedly induces cellular apoptosis through targeting the anti-apoptotic protein B-cell lymphoma-extra large (BCL-XL) in many cell types [86–88] as well as the IL-6/STAT3 pro-survival pathway [89]. While LIN28A/LIN28B represses apoptosis via let-7, it may also regulate the expression of pro-apoptosis and/or anti-apoptosis genes through unidentified mechanisms. For example, over-expression of LIN28B in oral cancer cells promotes the expression of Survivin, an apoptosis inhibitor [90].

Recently, the existence of a subclass of neoplastic cells, termed cancer stem cells (CSCs), in many tumors is thought to be the root cause of chemotherapeutic failure and tumor recurrence because CSCs are resistant to apoptosis [53]. While the LIN28A/LIN28B and let-7 loop is known to be involved in the development of chemotherapeutic sensitivity of cancer cells to apoptosis, it is also purportedly involved in the maintenance and/or differentiation of CSCs. Firstly, in many cancer types, high levels of LIN28A and other stem cell maintenance factors, such as OCT4, are present in a sub-population of cells with CSC properties [91–93]. Secondly, forced expression of LIN28A promotes the expression of CSC markers as well as the self-renewal capability of CSCs, while knock-down has the opposite effect [74, 92]. Lastly, through the down-regulation of let-7, enhanced expression of LIN28A induced the development of CSC ‘stemness’ coupled with resistance to chemotherapy-induced apoptosis [94, 95].

Of note, let-7 may inhibit apoptosis under certain conditions. For example, forced let-7a expression in A431 and HepG2 cells increased resistance to apoptosis induced by doxorubicin and paclitaxel through the direct targeting of caspase-3 [96]. Additionally, up-regulation of let-7 family miRNA expression upon estrogen exposure in endometrial adenocarcinoma enhanced cellular survival through the direct targeting of the anti-apoptosis gene BAX [97]. These results suggest that the let-7 family miRNAs play a multifaceted role in the regulation of cellular apoptosis.

### LIN28A/LIN28B and let-7 loop regulates genome instability

In normal cells, the stability and integrity of the genome is maintained by a functional DNA damage repair system. However, in cancer cells, this system is often defective resulting in genome instability and the accelerated accumulation of mutations. While these effects are typically necessary for and contribute to cancer progression, certain mutations may also be deadly for cancer cells or increase their sensitivity to various therapeutic modalities [53]. In radiation-treated cancer cells, LIN28A/LIN28B over-expression reportedly inhibits the expression of gamma-H2AX, which is an activate form of histone H2AX and is necessary for repair of double strand breaks (DSBs) [98], which suggests that LIN28A/LIN28B may increase genome instability by inhibiting DSB repair [85]. However, a recent study found that the expression of let-7 was decreased in colon cancer cells following radiation exposure [99]. Another study showed that p53 directly bound to and inhibited the expression of let-7 during this process [99]. Moreover, the exogenous expression of let-7 increased radiation-induced cytotoxicity, which suggests that let-7 family miRNAs may also increase the genome-instability of cancer cells.

### LIN28A/LIN28B and let-7 loop may regulate other hallmarks of cancer

Angiogenesis is required for tumors to survive as they need a steady supply of nutrients and oxygen as well as a means of evacuating metabolic waste. The most thoroughly-studied inducer of angiogenesis is vascular endothelial growth factor (VEGF). A recent study showed that stable expression of LIN28B in oral cancer cells promoted the expression of VEGF, suggesting that LIN28A/LIN28B may be involved in the regulation of tumor angiogenesis [90].

To generate macroscopic tumors, cancer cells also have to acquire the capability of replicative immortality, partially through conquering senescence, a barrier to proliferation and characterized as irreversible entrance into a non-proliferative but viable state [53]. Cellular senescence involves transcriptional repression of proliferation-promoting genes mediated by the retinoblastoma (RB1)/E2F transcriptional repressor complex. Interestingly, let-7 reportedly triggers human cell senescence through modifying chromatin at the promoters of RB1/E2F target genes, thus repressing their transcription, which suggests that the LIN28A/LIN28B and let-7 loop may also be involved in the regulation of cancer cellular replicative immortality [100].

### Crosstalk between LIN28/let-7 loop and oncogenes in regulating hallmarks of cancer

Transcription factor myc is a well-established oncogene. More than 70 % of all tumors have some form of c-MYC gene dysregulation [101]. It has been well-known that myc controls the proliferation of cancer cells. Recently, it has also been shown that myc regulates the metabolism, cancer related inflammation, metastasis, angiogenesis, and genome instability of malignancies. Thus, myc is a master regulator to control the progression of malignancies via mediating crosstalk of hallmarks of cancer.

The function of myc in regulating the proliferation of cancer cells has been well-documented. Myc directly transactivates most of the critical positive cell cycle regulators (such as Cdks and cyclins) but block the transcription of cell cycle inhibitors (such as p21). Moreover, myc directly promotes DNA replication by facilitating the replication initiation and hyperactivatescyclin/Cdk complexes via activation of Cdc25 phosphatases and Cdk activating kinase (CAK) [102]. In addition to regulate proliferation, the myc oncogene was shown to enhance glycolysis and alter amino acid metabolism in cancer cells [103]. Myc is known to directly activate the expression of almost all genes encoding glycolytic enzymes, such as lactate dehydrogenase (LDH), hexokinase 2 and enolase 1. Also, myc enhances glucose uptake via activating the expression of the glucose transporter GLUT1 [103, 104]. Besides glucose, cancer cells also take up and use glutamine to accumulate biomass. Glutamine is converted into glutamate by glutaminase (GLS), and glutamate drives the biogenesis of acetyl-CoA through a reverse TCA cycle. Myc promotes glutaminolysis and the generation of glutamate through activating the expression of GLS. As previously states, the activation of tumor associated macrophage (M2 type macrophage) showed pro-tumorigenic behavior. The data from Pello et al. demonstrated that the activation of tumor-associated macrophage requires the transcription factor c-MYC, and c-MYC controls the induction of about 45 % of genes associated with M2 macrophage activation, such as SCARB1, ALOX15 and MRC1; whereas myc inhibition prevents the activation of M2 macrophages and their pro-tumorigenic behavior [105]. Myc also regulates metastasis of malignancies [106]. It not only promotes EMT of cancer cells through activation of SNAIL and HMGA2, but also facilitates the invasion and migration of cancer cells via directly activating transcription of a bunch of invasion or migration-promoting factors, such as LGALS1, OPN and RhoA [106]. It has been demonstrated that myc is essential for the vasculogenesis and angiogenesis during tumor progression [107]. The mechanisms of myc masterly regulating angiogenesis was associated with that myc directly activates the expression of VEGF, a potent angiogenesis inducer, but indirectly inhibits the expression of angiogenesis inhibitors thrombospondin-1 through induction of miR-17-92 cluster [108]. Additionally, myc induced genome instability has been noticed recently [101]. Myc was revealed to affect genome amplifications, nucleus organization [101] and impair DNA damage repairs [109, 110].

**Fig. 4:**
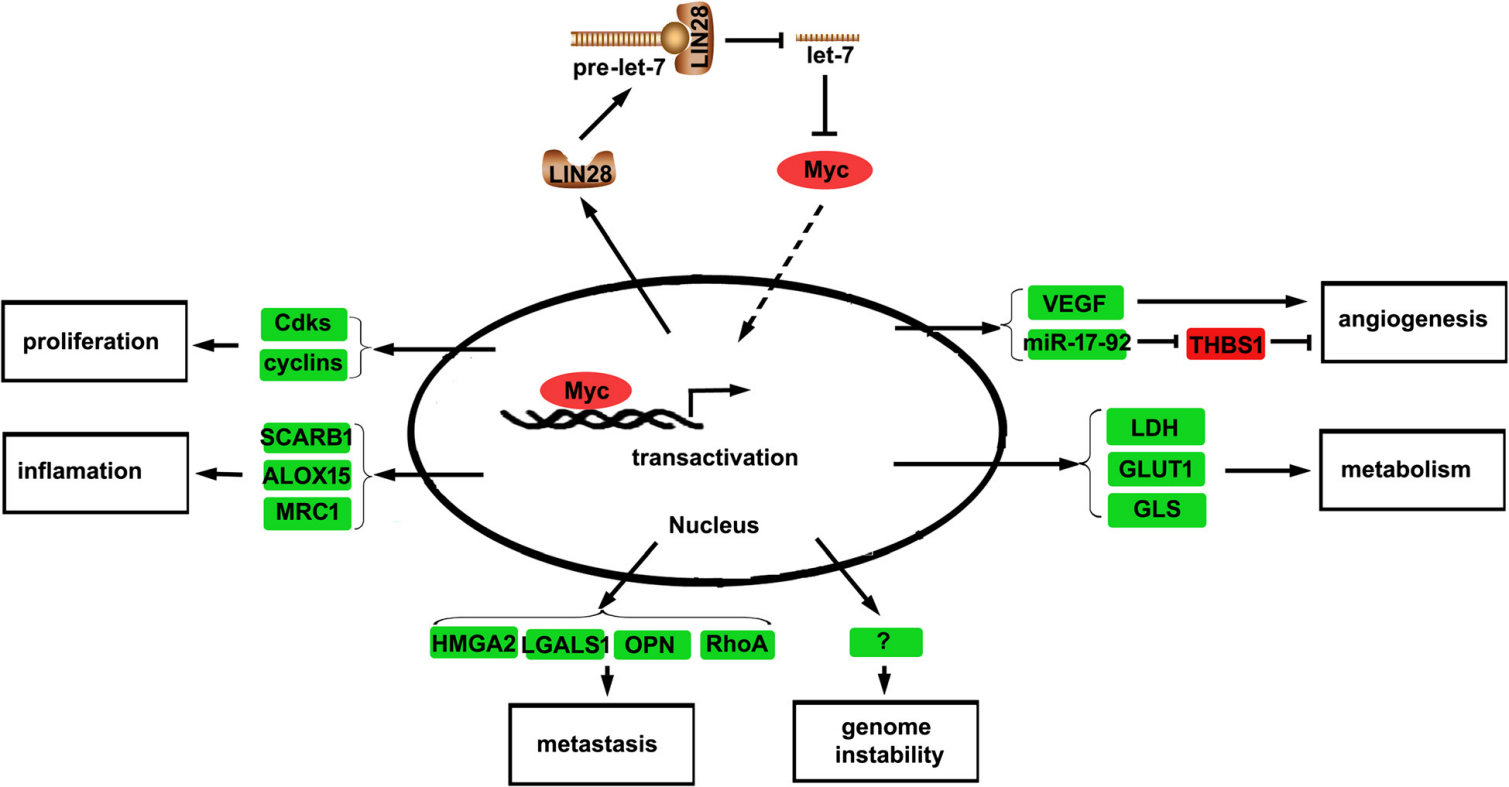
The LIN28/let-7/MYC feedbacks loop and the crosstalk of hallmarks of cancer

It has been revealed that c-myc can directly bind the promoter of LIN28B and thus elevate the production of LIN28B and consequently inhibit the generation of let-7 family of miRNAs upon activation of MAPK signaling [26]. Interestingly, over-expression of LIN28 was shown to elevate the expression of myc via down-regulation of let-7, which targets the MYC gene. These results suggested a complicated feedback loop consisting of LIN28B, let-7 and MYC. Since myc is one of the target genes of let-7, let-7-mediated inhibition of myc thus inhibits the crosstalk of hallmarks of cancers; LIN28A/LIN28B, of course, has the opposite effect. The LIN28/let-7/MYC feedbacks loop and the crosstalk of hallmarks of cancer has been shown in Fig. 4.

**Fig. 5:**
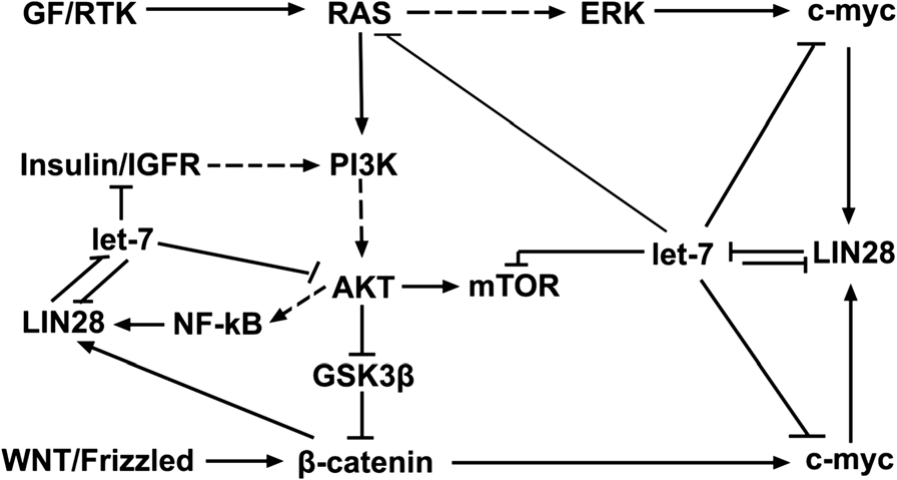
Crosstalk between LIN28/let-7 loop and oncogenes in regulating hallmarks of cancer

The expression of LIN28/let-7/MYC feedbacks is regulated by many signaling pathways and oncogenes. When Wnt signals are absent, the cytoplasm β-catenin is degraded by APC/GSK3β/Axin complex, while Wnt ligands bind to Frizzled receptor, cytoplasm β-catenin is accumulated and translocates to the nucleus where β-catenin promotes the transcription of myc gene [111]. This implies that β-catenin may also indirectly up-regulate LIN28 expression via elevating myc level. Recently, it has been reported that β-catenin can directly promote the transcription of LIN28B [29]. Growth factors binding to their receptors result in the activation of RAS, which either activates ERK (MAPK signaling) or PI3K/AKT signaling. ERK directly activates the transcription of myc, while AKT indirectly promotes the expression of myc gene via activating β-catenin activity [111], which suggests that RAS may regulate the expression of LIN28. Indeed, RAS has been found to inhibit the generation of let-7 by upregulating the expression of LIN28 via MAPK activated myc expression [26]. AKT also activates the NF-κB signaling via activating IKK, and NF-κB has been reported to directly promotes the transcription of LIN28B and thus inhibits the generation of let-7 s [27]. Interestingly, RAS and AKT are the direct targets of let-7 s respectively [60, 112]. These results suggested that there is a complicated crosstalk between RAS, PI3K/AKT, NF-κB, LIN28A/LIN28B and let-7 loop. The crosstalk between these oncogenes and LIN28A/LIN28B and let-7 loop is summarized in Fig. 5.

## Conclusion and perspective

In summary, in a variety of cancer types, let-7 is most frequently down-regulated, while LIN28A/LIN28B is most frequently up-regulated, and the aberrant expression of one component of theLIN28A/LIN28B and let-7 loop due to transcriptional and/or post-transcriptional level dysregulation in human malignant tumors would result in the alteration of the other one. High levels of LIN28A/LIN28B and low levels of let-7 contribute to the development of human malignances through promoting cellular proliferation, cell death resistance, angiogenesis, metastasis, metabolism reprogramming, tumor-associated inflammation, genome instability, acquiring immortality and evading immune destruction of cancer cells. The many established studies suggest that the LIN28A/LIN28B and let-7 loop is a master regulator of cancer development and would be a valuable target for future cancer therapeutic strategies.
